# Willingness to pay for and willingness to vaccinate with the COVID-19 vaccine booster dose in China

**DOI:** 10.3389/fphar.2022.1013485

**Published:** 2022-09-20

**Authors:** Hui Jun Zhou, Lei Pan, Hui Shi, Ji Wei Luo, Pei Wang, Hannah K. Porter, Ye Bi, Minghui Li

**Affiliations:** ^1^ Department of Public Administration, Business School, University of Shanghai for Science and Technology, Shanghai, China; ^2^ Department of Orthopedics, Zengcheng Branch, Nanfang Hospital, Southern Medical University, Guangzhou, China; ^3^ School of Public Health, Fudan University, Shanghai, China; ^4^ Key Lab of Health Technology Assessment, National Health Commission, Fudan University, Shanghai, China; ^5^ College of Pharmacy, Univesity of Tennessee Health Science Center, Memphis, TN, United States; ^6^ I.Baby Preconception Care, Shanghai, China; ^7^ Department of Clinical Pharmacy and Translational Science, Univesity of Tennessee Health Science Center, Memphis, TN, United States

**Keywords:** willingness to pay, willingness to vaccinate, COVID-19, vaccine, booster, health belief model, iterative bidding game

## Abstract

**Objective:** The present study aims to assess the willingness to pay (WTP) for and willingness to vaccinate (WTV) with the Coronavirus (COVID-19) vaccine booster dose in China when the pandemic is under adequate control and the majority of the population is vaccinated. This study is also to identify significant factors associated with the WTP.

**Methods:** This was a cross-sectional study on adults with no past or present COVID-19 infection. An online questionnaire was distributed to collect data on vaccination status, quarantine experience, and factors related to health beliefs on vaccination. The WTV was assessed through the vaccination preference. The WTP was examined by payment scale (PS) and iterative bidding game (IBG) administered in random order. Three IBG algorithms with different starting-price were presented randomly. The average WTP of PS and IBG were analyzed as primary outcomes using univariate and multivariate analyses. Multivariate ordered logistic regression was performed to identify significant factors for the WTP.

**Results:** The survey recruited 543 participants with a mean age of 32 years and 57.80% being female. The WTV rate was 86.74%, while 94.66% of participants completed full-schedule or enhanced vaccination. The mean WTP was CNY 149 (±CNY 197) and the median WTP was CNY 80. Regarding significant factors for the WTP, urban residents were 57% more likely (95% CI: 1.11-2.22) to pay for a high-priced vaccine than rural residents. Respondents who completed full-schedule vaccination were 46% more likely (95% CI: 1.03–2.07) to pay for a high-priced vaccine than those who completed enhanced vaccination. Respondents with a low household income of CNY 40k or lower were 62% less likely (95% CI: 0.21–0.66) to pay for a high-priced vaccine than those with a middle household income of CNY 110k–210k. Other significant factors associated with the WTP included the perceived benefit of vaccination and peer environmental pressure in the health belief model.

**Conclusion:** The WTV with the COVID-19 vaccine booster dose was high in China. The WTP was influenced by the place of residence, vaccination status, household income, perceived benefit of vaccination, and environmental peer pressure. Study findings can inform policymakers to better design vaccination programs and financial schemes involving out-of-pocket payments.

## 1 Introduction

The Coronavirus (COVID-19) pandemic continues to be a global public health crisis and has caused huge economic and health damage worldwide. Mass vaccination aiming for herd immunity has been adopted as a national strategy in many countries to protect the population from being infected or developing severe conditions (Ayifah and Ayifah, 2022). Since December 2020, China has launched two rounds of vaccination programs and has been actively promoting the COVID-19 vaccine booster dose (GotPsRo, 2021). Domestically-manufactured vaccines by SinoVac or SinaPharm are provided free of charge to all citizens in light of the zero-COVID policy. Herd immunity of 75%–90% vaccination coverage was obtained in China, as well as in many other countries around the world (Anderson et al., 2020).

The effect of initial mass vaccination is limited as reflected by multiple COVID-19 resurgences worldwide and the recent outbreak in Shanghai. Immunity to COVID-19 can be undermined by the waning effect of vaccination, evolving variants, and virus breakthroughs. To cope with this challenge, the booster dose has been utilized by health authorities. Experts proposed an annual booster dose as a long-term strategy to control cross-border transmission and local outbreaks.

However, a long-term vaccination program is challenging both financially and socially. Providing vaccinations as public goods to a huge population is costly for the government given the current economic decline. Chinese National Healthcare Security Administration announced that the National Medical Fund would no longer subsidize routine nucleic acid amplification tests (Administration, 2022). Copayments or complete out-of-pocket charges may become a requirement in the future in order to sustain vaccination needs.

Vaccine hesitancy has been the major reason for the inability to control the COVID-19 infection (Iyer et al., 2022). Studies found that the success of long-term vaccination is closely related to the willingness to pay (WTP) for and willingness to vaccinate (WTV) against public viruses (Wane et al., 2019; Lai et al., 2020). WTP informs the maximum amount of money a customer is willing to pay for a specific good based on personal valuation and is commonly estimated using contingent valuation methods (CVM). WTV indicates the vaccination intention which can be used to predict actual vaccination behavior. The evidence surrounding WTP and WTV has assisted in policy development, vaccine pricing, government purchasing, and program design (Hynes et al., 2021). At the beginning of the COVID-19 epidemic, WTV and WTP were investigated in the Chinese population. Studies have reported the median WTP for COVID-19 to be CNY 100, 200, or 300 (Wang et al., 2021; Lin et al., 2020; Han et al., 2021) and the mean WTP to be CNY 130.45 and 254 (Qin et al., 2021; Wang et al., 2021). A higher price range of CNY 501–1,000 was once reported as the most preferred price for the general Chinese population (Zhang et al., 2021). On the other hand, WTV rates were estimated to be 83.5%, 77.4%, and 89.1% (Lin et al., 2020; Zhang et al., 2021; Han et al., 2021). Both WTP and WTV were largely affected by socioeconomic variables and variables measuring personal health beliefs, such as perceived risk and perceived benefit of vaccination in line with the health belief model (HBM) (Goruntla et al., 2021).

WTP and WTV varied with the severity of the epidemic (Wang et al., 2022). Early studies were conducted prior to the introduction of the COVID-19 vaccine to the market, therefore the findings may not bear much value to guide future vaccination policy. Now that the target population has been vaccinated and has personally experienced the effect of receiving or abstaining from vaccination, attitudes surrounding WTP and WTV were anticipated to change.

We hypothesized that an annual booster dose of the COVID-19 vaccine will become a national strategy in China for the next several years and that supplying vaccines as public goods may not be sustainable. WTP and WTV change over time, therefore reevaluation is required to inform the feasibility of an alternative financing scheme, as well as program design and adaptation. This study was conducted aiming to assess the WTP and WTV of the general Chinese population for the COVID-19 vaccine booster dose. This study is also designed to identify the significant factors contributing to the WTP.

## 2 Materials and methods

### 2.1 Study design

This cross-sectional study was conducted through the largest online survey platform in China, Wen Juan Xing (Changsha Ranxing Information Technology Co., Ltd., Hunan, China). Wen Juan Xing is equivalent to Qualtrics, SurveyMonkey, or CloudResearch and provides online questionnaire design and survey functions for customers. The questionnaire was posted in January 2022 untill March 2022. Participants were allowed to answer the questionnaire through individual WeChat accounts only once in anonymity. Snowballing sampling was adopted and started with a convenience sample composed of colleagues, friends, and their families. The questionnaire was then circulated *via* the existing respondents. The inclusion criteria were broadly defined, 1) ≥18 years, 2) no history of COVID-19 infection, and 3) ability to read Chinese. The survey was voluntary, and no incentive was offered.

### 2.2 Data collection

#### 2.2.1 Sample characteristics

The questionnaire inquired respondents about demographic information, socioeconomic status [highest education, marital status, annual household income (AHI), place of residence, medical insurance, etc.], health status (self-rated health status, concurrent chronic diseases), and vaccine dose received. The impact of COVID-19 policies on personal life was explored by asking if respondents had been quarantined at home or in a hotel. Exposure to COVID-19 was measured by the question regarding the presence or absence of recent positive cases in the respondents’ community or workplace. Respondents also provided their experience of vaccination and infection.

#### 2.2.2 Health belief

As informed by the HBM theory (Lau et al., 2010), we adapted a previous Chinese HBM questionnaire to investigate the individuals’ beliefs in four dimensions with eight questions. These four dimensions included perceived susceptibility (“Infection with COVID-19 is possible for me at present,” “The probability of infection is high for me for the next few months”), perceived severity (“I will be very sick if I got COVID-19 infection”), perceived benefits (“Vaccination will decrease my risk of getting an infection or developing severe complications if infected”), and perceived barriers (“I am concerned about the effectiveness of the COVID-19 vaccine,” “I am concerned about the safety of the COVID-19 vaccine,” “I am concerned about my affordability considering the cost of vaccination”). One question was dedicated to measuring environmental peer pressure (“I will accept vaccination if others accept it”). Each question was assigned four options, “strongly agree,” “agree,” “disagree” or “strongly disagree” to be consistent with the previous questionnaire (Lin et al., 2020).

#### 2.2.3 Willingness to vaccinate assessment

The WTV in our study is the willingness to receive the COVID-19 vaccine booster dose given respondents have been vaccinated, witnessed the impact of COVID-19 policies, and generally understood the scope and severity of the current COVID-19 epidemic. The WTV question was “Will you accept the COVID-19 vaccine in the future if vaccination is required.” The extent of the agreement was ordered as “definitely yes,” “probably yes,” “not sure,” “probably no,” and “definitely no.” The “definitely no” precludes the subsequent WTP questions and was taken as zero.

#### 2.2.4 Willingness to pay assessment

The WTP in our study examined the maximum amount a person was willing to pay for the booster dose of the COVID-19 vaccine. Based on the CVM methodology, payment scale (PS) and iterative bidding game (IBG) were developed to elicit the stated WTP. Two methods were used in parallel to mitigate the bias inherent in either method (Frew et al., 2004). Price ranges for WTP were derived from the purchase price of category-2 vaccines (out-of-pocket payment and voluntary vaccination), 10% of national monthly income, and medical expense per capita in China from 2016 to 2020 (Catma and Varol, 2021; Ayifah and Ayifah, 2022). A range of CNY 20–800 was deemed reasonable and was applied to both PS and IBG. In order to avoid the artificial between-method difference in WTP solely caused by price cutoffs, the price strata were set consistently for PS and IBG. PS used 10 strata, CNY 20, 80, 110, 140, 170, 200, 350, 425, 500, and 800, while IBG used nine bids, i.e., CNY 20, 80, 140, 170, 200, 350, 425, 500, and 800. Whether for PS or IBG, an open-ended question was followed if a respondent chosed the highest price of CNY 800.

**FIGURE 1:**
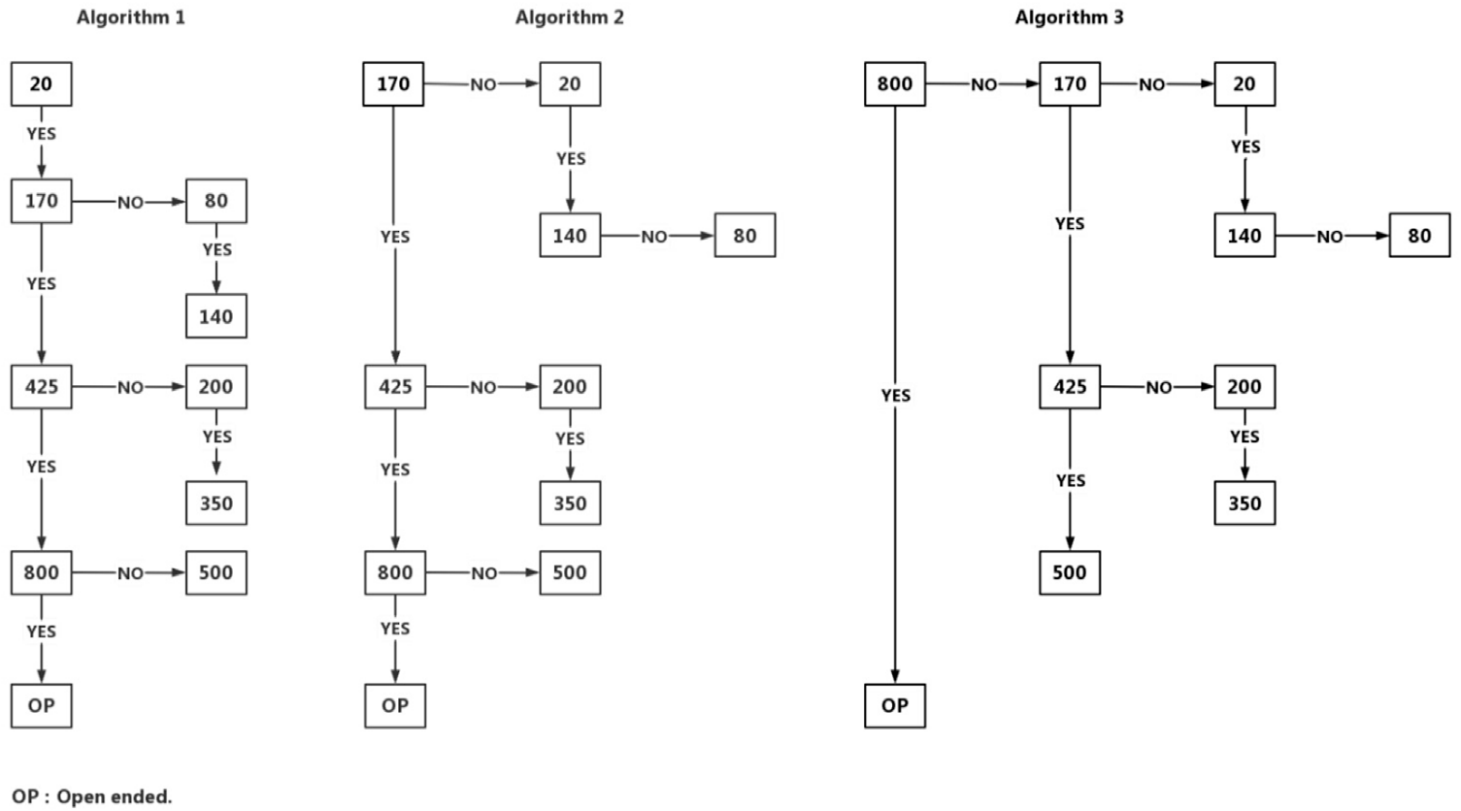
Iterative bidding game algorithms.

The PS simply asked the correspondents to choose one of 10 preset prices to indicate their WTP or provide an amount if their WTP is above CNY 800. The IBG first gave a brief update regarding the epidemic, prevailing policies, and social effect of COVID-19 vaccination (Frew et al., 2010). Following the update, an usher-in question was presented that stated “Individuals should pay for COVID-19 vaccine out-of-pocket” with five options: “absolutely correct,” “probably correct,” “not sure,” and “probably wrong,” and “absolutely wrong.” Those answering “absolutely wrong” did not proceed to the subsequent IBG algorithms. Accordingly, their WTP were marked as zero. Three IBG algorithms, IBG20, IBG170, and IBG800, were designed with initial bids of CNY 20, 170, and 800 respectively, to minimize the starting-price bias or anchoring effect (Figure 1) (Frew et al., 2004). Each IBG algorithm would lead to seven ending prices of CNY 20, 80, 140, 200, 350, 500, and 800. Additionally, the respondents were given a chance to state their maximum WTP if it is over CNY 800.

To minimize the information bias due to the ordering effect in estimation, random allocation procedures were taken in two steps. When a respondent was starting to answer WTP questions, one CVM method (PS or IBG) was randomly assigned first and then followed by the other. When it came to IBG algorithms, respondents were instructed to randomly pick a number between 1, 2, and 3, corresponding to three IBG starting prices, and next move forward to complete the bidding process. As a result, the respondents were randomly assigned to six pathways, IBG20-PS (8.17%), IBG170-PS (18.24%), IBG800-PS (0.87%), PS-IBG20 (34.47%), PS-IBG170 (24.65%) and PS-IBG800 (12.97%).

#### 2.2.5 Statistical analysis

A total of 545 subjects participated in the study by March 2022. Questionnaires of two respondents (COVID-19 positive) were removed for the reasons of ineligibility or missing information. Descriptive statistics were performed to summarize the continuous variables with a mean (standard deviation, SD) and categorical variables with a number (proportion). Univariate analyses included *t*-test, ANOVA, and Chi-square test to carry out comparisons. The WTP derived from PS and IBG were subjected to an agreement test capturing an absolute intra-class correlation coefficient of 0.132, which indicated little agreement of PS with IBG estimates (Koo and Li, 2016). Therefore, the WTP-PS and WTP-IBG were averaged, and the WTP-average was analyzed as the primary outcome.

As the distribution of WTP-average was highly skewed to the right, it was categorized into five levels, CNY 0, 0–80, 81–200, 201–500, >500, with referencing to benchmarks and the parameters of distribution (null or mode, median, mean, 75%, and 95% percentile). Setting the categorical WTP-average as the dependent variable, multivariate ordered logistic regressions were performed to identify the significant predictors of WTP. There were 10 subjects giving extremely opposite WTPs by PS and IBG (e.g., maximum WTP in PS yet minimum WTP in IBG, or vice versa). These data were considered invalid and therefore excluded from the WTP analysis. The analyses were performed with SPSS 26. A *p* < 0.05 was taken as statistically significant.

## 3 Results

### 3.1 Characteristics of the sample

The mean age of the sample (*n* = 543) was 32 years with 36 (6.60%) subjects older than 50 years (Table 1). There were more females (57.80%) and urban residents (52.12%). Families ranked in the middle class (AHI from CNY 110 to 210k) accounted for 46.22%. The number of participants from relatively poor families (*n* = 67, AHI < CNY 40k) was almost equal to those from super rich families (*n* = 66, AHI > CNY 450k). Twenty-six (4.78%) persons were not covered by any medical insurance. The majority (89.32%) rated their health good or very good, while 5.89% of the sample had self-reported chronic conditions. There were 96 (17.86%) participants having been quarantined at home and another 15 (2.76%) persons having been quarantined in a hotel. A total of 56 (10.31%) persons have been exposed to either community or workplace infection recently.

**TABLE 1:** Characteristics of study participants and willingness to vaccinate.

Variable	Category	N	Percent (%)
Age (years)	18–24	209	38.49
	25–35	161	29.65
	36–50	137	25.23
	>50	36	6.63
Gender	Male	229	42.17
	Female	314	57.83
Occupation	Professional	124	22.84
	Company staff	90	16.57
	College students and below	96	17.68
	Graduate students and above	121	22.28
	Others	112	20.63
Marital status	Married/Divorced	228	41.99
	Single	315	58.01
Highest education level	Junior college or below	120	22.10
	Bachelor’s degree	243	44.75
	Master’s degree or above	180	33.15
Place of residence	Urban	260	47.88
	Rural	283	52.12
Annual household income (CNY 1,000)	≤40	67	12.34
	40–70	73	13.44
	70–110	122	22.47
	110–210	129	23.76
	210–450	86	15.84
	>450	66	12.15
Chronic diseases	No	511	94.11
	Yes	32	5.89
Health status self-rated	Very good	224	41.25
	Good	261	48.07
	Fair/Poor/Very poor	58	10.68
Medical insurance	No	26	4.79
	National medical insurance for urban employees	168	30.94
	National medical insurance for urban residents and rural citizens	164	30.20
	National medical insurance and other insurance	127	23.39
	Other insurance	58	10.68
Quarantine experience	Hotel quarantine	17	3.13
	Home quarantine	97	17.86
	No	428	78.82
Recent exposure to COVID-19	No	487	89.69
	Yes	56	10.31
Actual vaccination status	One shot	29	5.34
	Two shots	188	34.62
	Three shots	326	60.04
Willingness to vaccinate	Definitely yes	325	59.85
	Probably yes	146	26.89
	Not sure/Probably no/Definitely no	72	13.26
Out-of-pocket payment for vaccine	Absolutely correct	28	5.20
	Probably correct	116	21.70
	Not sure	249	46.60
	Probably wrong	52	9.70
	Absolutely wrong	89	16.70
Perceived current risk of infection	Strongly agree/Agree	78	14.36
	Disagree	172	31.68
	Strongly disagree	293	53.96
Perceived short-term risk of infection	Strongly agree/Agree	43	7.92
	Disagree	222	40.88
	Strongly disagree	278	51.20
Perceived severity of infection	Strongly agree/Agree	71	13.08
	Disagree	245	45.12
	Strongly disagree	227	41.80
Perceived benefit of vaccination	Strongly agree	212	39.04
	Agree	277	51.01
	Strongly disagree/Disagree	54	9.94
Concerned about vaccine efficacy	Strongly agree/Agree	178	32.78
	Disagree	267	49.17
	Strongly disagree	98	18.05
Concerned about vaccine safety	Strongly agree/Agree	114	20.99
	Disagree	309	56.91
	Strongly disagree	120	22.10
Environmental peer pressure	Strongly agree/Agree	196	36.10
	Disagree	240	44.20
	Strongly disagree	107	19.71
Concerned about vaccination cost	Strongly agree/Agree	172	31.68
	Disagree	265	48.80
	Strongly disagree	106	19.52

CNY, Chinese Yaun currency; COVID-19, Coronavirus disease 2019.

### 3.2 Health belief assessment

As presented in Table 1, the risk perception of COVID-19 in our sample was low, as only 14.36% and 7.92% of the respondents agreed with the current or short-term risk of infection. Only 71 (13.08%) respondents agreed that COVID-19 was a severe disease, while 489 (90.06%) participants were in agreeance with the protection of the vaccine. The efficacy and safety of the COVID-19 vaccine still concerned 32.78% and 20.99% of the sample respectively. Vaccination cost became an issue for 31.68% of the sample, and 196 (36.10%) participants would accept the vaccination only if others accepted it.

### 3.3 Willingness to vaccinate and willingness to pay

All participants were vaccinated, and the rates of full-schedule and enhanced vaccination totaled 94.66%. However, the WTV (“Definitely yes” or “probably yes” to vaccination) rate was comparatively lower at 86.74% (Table 1). Still, 9 (1.66%) and 17 (3.13%) subjects chose “definitely not” or” probably not” to the booster dose.

**FIGURE 2:**
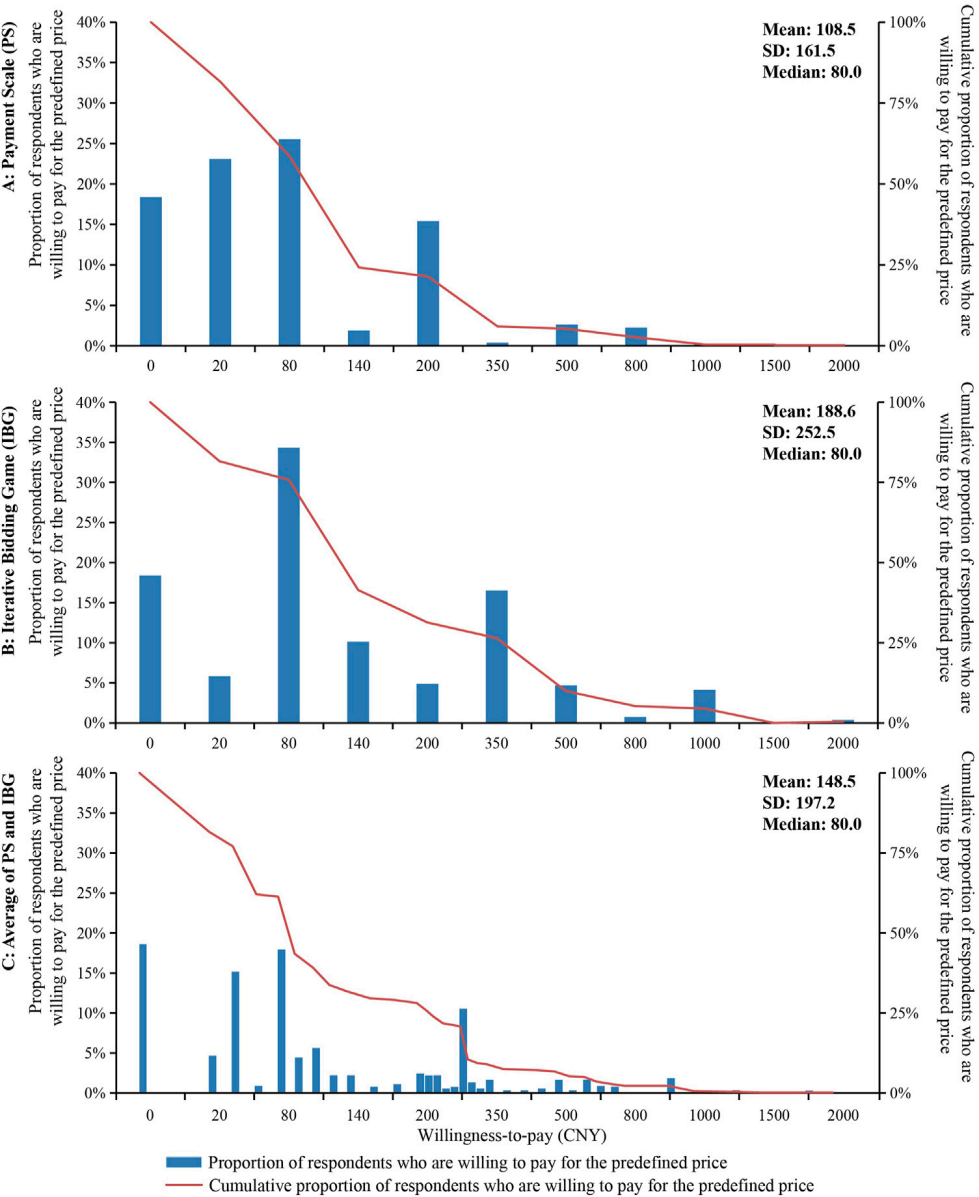
Willingness to pay distributions and demand curves of COVID-19 vaccine booster dose.

The distributions of WTP and the demand curve of the COVID-19 vaccine were presented in Figure 2. In consistency with the theory, the WTP prices are distributed to the right irrespective of the estimation approach. PS and IBG derived the same median and mode for WTP, which both were CNY 80. IBG derived a significantly higher mean WTP of CNY 189 than PS (mean = CNY 109). The WTP-average captured more intermediate WTPs with the median and mean being CNY 80 and 149 respectively.

### 3.4 Significant factors for willingness to pay

The WTP-average was presented in Table 2 for different groups. Univariate analyses revealed that gender, place of residence, AHI, chronic disease, actual vaccination status, concerns about safety and cost of vaccination, actual vaccination status, and WTV were significant factors for WTP through between-group comparisons. Specifically, males and urban residents were willing to pay more than their counterparts. The WTP increased with the AHI. Chronic conditions predisposed a person to pay CNY 85 more for the vaccine. Vaccination predisposed a person to pay less, as those receiving the booster shot preferred a price of CNY 129, notably lower than those receiving one or two shots only. The WTV had a positive relationship with the WTP. “Definitely yes” or “probably yes” to WTV were associated with mean WTPs of CNY 156 and 165, which was higher than the mean WTP (CNY 85) for the group “not sure,” “probably no” or definitely no.” For HBM variables, respondents who were not concerned about the vaccine safety or vaccination cost were willing to pay CNY 172 and CNY 170 respectively, which was higher than those who were concerned about these issues.

**TABLE 2:** Willingness to pay in different groups.

Variable	Category	n	WTP-Average (CNY)			
			Median	Mean	S.D.	P
Age (years)	18–24	206	80	147	208	0.826
	25–35	156	80	148	175	
	36–50	135	80	158	213	
	>50	36	80	124	163	
Gender	Male	222	80	171	218	0.028
	Female	311	80	133	180	
Occupation	Professional worker	120	80	152	210	0.629
	Company staff	89	95	166	189	
	College students and below	95	80	163	254	
	Graduate students and above	118	80	131	140	
	Others	111	80	137	187	
Marital status	Married/Divorced	225	80	149	198	0.969
	Single	308	80	148	197	
Highest education level	Junior college or below	119	80	152	198	0.857
	Bachelor degree	239	80	152	216	
	Master’s degree or above	175	80	142	169	
Place of residence	Urban	278	80	170	219	0.008
	Rural	255	80	125	168	
Annual household income (CNY 1,000)	≤40	64	50	86	112	0.001
	40–70	72	80	120	158	
	70–110	120	80	142	169	
	110–210	129	95	148	158	
	210–450	85	80	166	199	
	>450	63	80	235	345	
Chronic diseases	No	503	80	144	181	0.022
	Yes	30	95	229	370	
Health status self-rated	Very good	217	80	144	197	0.819
	Good	259	80	154	202	
	Fair/Poor/Very poor	57	80	142	176	
Medical insurance	No	25	50	88	104	0.082
	National medical insurance for urban workers	164	80	166	218	
	National medical insurance for urban and rural residents	160	80	124	137	
	National medical insurance and other insurance	127	80	153	185	
	Other insurance	57	80	185	300	
Quarantine experience	Hotel quarantine	15	80	199	278	0.586
	Home quarantine	96	80	152	229	
	No	422	80	146	186	
Recent exposure to COVID-19	No	478	80	146	195	0.334
	Yes	55	80	173	217	
Actual vaccination status	One shot only	29	80	224	330	0.008
	Two shots	183	95	171	202	
	Three shots	321	80	129	176	
Willingness to vaccinate	Definitely yes	316	80	156	214	0.011
	Probably yes	145	80	165	189	
	Not sure/Probably no/Definitely no	72	50	85	102	
Perceived current risk of infection	Strongly agree/Agree	78	80	170	239	0.554
	Disagree	169	80	147	195	
	Strongly disagree	286	80	143	186	
Perceived short-term risk of infection	Strongly agree/Agree	42	80	145	160	0.310
	Disagree	218	80	164	219	
	Strongly disagree	273	80	137	183	
Perceived severity of infection	Strongly agree/Agree	70	80	195	242	0.087
	Disagree	240	80	147	195	
	Strongly disagree	223	80	136	182	
Perceived benefit of vaccination	Strongly agree	208	80	157	196	0.369
	Agree	273	80	149	202	
	Strongly disagree/Disagree	52	50	114	178	
Concerned about vaccine efficacy	Strongly agree/Agree	175	80	137	207	0.163
	Disagree	264	80	164	202	
	Strongly disagree	94	80	125	161	
Concerned about vaccine safety	Strongly agree/Agree	112	80	123	165	0.006
	Disagree	305	80	172	223	
	Strongly disagree	116	80	112	139	
Environmental peer pressure	Strongly agree/Agree	195	80	162	216	0.067
	Disagree	235	80	155	197	
	Strongly disagree	103	50	108	153	
Concerned about vaccination cost	Strongly agree/Agree	170	80	134	153	0.033
	Disagree	261	80	170	231	
	Strongly disagree	102	65	116	159	

CNY, Chinese Yaun currency; COVID-19, Coronavirus disease 2019.

When all variables were submitted to the multiple ordered logistic model, five factors were significant including two socioeconomic variables (place of residence and AHI), two HBM variables (perceived benefit of vaccination and peer environment pressure), and actual vaccination status (Table 3). Urban residents were 57% more likely (95% CI: 1.11–2.22) to pay for a high-priced vaccine than rural residents. Respondents with a low household income of CNY 40,000 or lower were 62% less likely (95% CI: 0.21–0.66) to pay for a high-priced vaccine than those with a middle household income of CNY 10,000–CNY 210,000. Compared to those who completed enhanced vaccination, respondents who completed full-schedule vaccination were 46% more likely (95% CI: 1.03–2.07) to pay for a high-priced vaccine. Respondents who do not see the perceived benefit of vaccination were 51% less likely (95% CI: 0.26–0.95) to pay for a high-priced vaccine than those who see the perceived benefit of vaccination. Respondents who do not have peer environmental pressure were 52% less likely (95% CI: 0.26–0.87) to pay for a high-priced vaccine than those who have peer environmental pressure.

**TABLE 3:** Factors associated with the willingness to pay a high price for the COVID-19 vaccine booster dose.

Variables	Categories	Ordered logistic regression for WTP		
		Odds ratio	95% confidence interval	*p*-Value
Age (years)	18–24	1	—	—
	25–35	1.26	0.75–2.11	0.392
	36–50	1.56	0.72–3.39	0.259
	>50	0.99	0.37–2.66	0.982
Gender	Male	1.22	0.86–1.73	0.262
	Female	1	—	—
Occupation	Professional worker	1	—	—
	Company staff	1.75	1.01–3.03	0.045
	College students and below	1.46	0.72–2.98	0.293
	Graduate students and above	1.43	0.65–3.17	0.374
	Others	1.13	0.64–2.00	0.665
Marital status	Married/Divorced	0.55	0.28–1.07	0.078
	Single	1	—	—
Highest education level	Junior college or below	1	—	—
	Bachelor degree	0.88	0.51–1.53	0.657
	Master’s degree or above	0.67	0.29–1.54	0.344
Place of residence	Urban	1.57	1.11–2.22	0.012
	Rural	1	—	—
Annual household income (CNY 1,000)	<40	0.38	0.21–0.66	0.001
	40–70	0.58	0.34–1.02	0.057
	70–110	0.73	0.46–1.18	0.200
	110–210	1	—	—
	210–450	0.82	0.48–1.40	0.466
	>450	0.91	0.50–1.68	0.770
Chronic diseases	No	1	—	—
	Yes	1.17	0.57–2.41	0.669
Health status self-rated	Very good	1.22	0.68–2.20	0.500
	Good	1.36	0.78–2.36	0.281
	Fair/Poor/Very poor	1	—	—
Medical insurance	No	0.5	0.22–1.14	0.097
	National medical insurance for urban workers	1	—	—
	National medical insurance for urban and rural residents	1.09	0.67–1.77	0.723
	National medical insurance and other insurance	1.09	0.67–1.80	0.721
	Other insurance	1.08	0.61–1.90	0.793
Quarantine experience	Hotel quarantine	0.75	0.26–2.14	0.584
	Home quarantine	1.11	0.72–1.72	0.630
	No	1	—	—
Recent exposure to COVID-19	No	1	—	—
	Yes	1.49	0.89–2.48	0.126
Actual vaccination status	One shot only	1.47	0.64–3.41	0.368
	Two shots	1.46	1.03–2.07	0.032
	Three shots	1	—	—
Perceived current risk of infection	Strongly agree/agree	0.85	0.45–1.60	0.617
	Disagree	0.81	0.49–1.33	0.396
	Strongly disagree	1	—	—
Perceived short-term risk of infection	Strongly agree/Agree	1.35	0.60–3.02	0.470
	Disagree	1.46	0.88–2.43	0.141
	Strongly Disagree	1	—	—
Perceived severity of infection	Strongly agree/Agree	1.21	0.65–2.27	0.551
	Disagree	0.86	0.54–1.36	0.508
	Strongly Disagree	1	—	—
Perceived benefit of vaccination	Strongly agree	1	—	—
	Agree	0.83	0.57–1.20	0.311
	Disagree/Strongly disagree	0.49	0.26–0.95	0.036
Concerned about vaccine efficacy	Strongly agree/Agree	1	—	—
	Disagree	1.17	0.73–1.86	0.522
	Strongly disagree	1.23	0.59–2.57	0.586
Concerned about vaccine safety	Strongly agree/Agree	1	—	—
	Disagree	1.32	0.78–2.24	0.307
	Strongly disagree	1.06	0.47–2.42	0.885
Environmental peer pressure	Strongly agree/Agree	1	—	—
	Disagree	1.01	0.70–1.47	0.946
	Strongly disagree	0.48	0.26–0.87	0.017
Concerned about vaccination cost	Strongly agree/Agree	1	—	—
	Disagree	1.00	0.68–1.47	0.991
	Strongly disagree	0.82	0.45–1.50	0.525

CNY, Chinese Yaun currency; COVID-19, Coronavirus disease 2019.

## 4 Discussion

COVID-19 has had drastic economic, social, and public health implications, and continues to pose a significant threat worldwide. The COVID-19 vaccine booster dose has been implemented globally and is becoming a long-term public health safety measure. The WTP and WTV are important indicators of the population’s attitude toward continuous vaccination, and these measures fluctuate with the progression of the epidemic and its effect on policies. Therefore, continuous assessment of the WTP and WTV is of great significance. With this in mind, this study was conducted and found that the overall WTV rate was 86.74% in China given a 100% coverage rate and a 94.66% full vaccination rate. The median and mean WTPs were CNY 80 (USD 12.40) and CNY 149 (USD 23.02) respectively. Place of residence, AHI, vaccination status, perceived benefit of vaccination, and environmental peer pressure can significantly predict the concurrent WTP.

Our results appear encouraging, as both the actual vaccination rate and WTV are high. Compared to the WTV rates of 83.50%, 77.40%, and 89.10% in the early phase of the COVID-19 epidemic in China (Lin et al., 2020; Zhang et al., 2021; Han et al., 2021), the current WTV rate of 86.74% did not drop at a time that COVID-19 was under adequate control. Previous studies were conducted before the COVID-19 vaccine was available, and vaccination programs were not yet implemented. Theoretically, WTV at that time should have been higher because the strong wish to terminate the epidemic altogether would have predisposed more people to accept the vaccine. The WTV in our study represented the vaccination intention of vaccinated persons, who were more informed and realistic about the vaccination effect and witnessed the success of non-medical measures. The WTV rate remained satisfactory suggesting a promising prospect for the long-term vaccination strategy in China. As a developing country, the WTV rate of China was similar to that of other developing countries such as India and Kenya (Carpio et al., 2021; Goruntla et al., 2021), yet higher than that of developed countries such as Germany, the Netherland, and France (Neumann-Bohme et al., 2020).

Contrary to the theory and empirical evidence that the actual vaccination rate was always lower than the WTV rate (Wang et al., 2022), our study found the opposite. The full-schedule vaccination rate among our participants was 7.92% higher than the WTV rate. There were 61 subjects showing reluctance to vaccination, yet vaccinated anyhow. The high vaccination rate discovered in our study was generally consistent with the high acceptance of other personal protective measurements, such as mask-wearing and hand-washing in the Chinese population (Zhong et al., 2020). The extra vaccination above WTV indicates that external factors in addition to personal intention were taking effect. This is in line with the theory about the influence of external action plans on public willingness. An Indian study proved that governmental propagation of the COVID-19 vaccine enhanced the WTV rate significantly (Goruntla et al., 2021). This is most likely the case in China, as the Chinese government had implemented the zero-COVID policy, in contrast to “Live with COVID-19” in advanced countries (Kirby, 2022). Under the umbrella of the zero-COVID strategy, 1) vaccination was mandated to some institutionalized populations such as students and government officers (Ioannidis, 2021), 2) non-vaccination restricts people from essential activities like working in office or business traveling, 3) authorities provide powerful education about the severity and fatality of COVID-19 infection and benefit of vaccines (Zhang et al., 2021; Zhang et al., 2022), 4) some cities provided incentives to push for the vaccination, 5) last but not least, the vaccine is free. All these interventions would boost the vaccination rate as proven in other populations (Iyer et al., 2022).

Our study illustrated an expected, yet concerning, phenomenon that high vaccination status would dissuade a person from paying for a vaccine. The WTP estimated from our sample was lower than previously observed. The median WTP-average was CNY 80, below the median WTPs of CNY 100, 200, and CNY 300 previously reported for the Chinese population (Wang et al., 2021; Lin et al., 2020; Han et al., 2021). Likewise, the mean WTP in our study was CNY 149, lower than the mean WTPs of CNY 254 and 130 (Qin et al., 2021; Wang et al., 2021). One study published that the most preferred WTP range was CNY 501–1,000 (Zhang et al., 2021). Additionally, WTP was negatively associated with the number of shots. Full-schedule and booster-vaccinated persons would pay CNY 95 and 563, less than that of those who had received one shot only. This was further consolidated in multivariate analyses showing respondents boosted with the third shot were significantly reluctant to pay a higher price for the COVID-19 vaccine. This downward trend of WTP is not surprising in that the perceived current risk of infection was low at 14.36%, and the perceived risk in the next months was even lower at 7.92%. Studies have already shown that higher perceived risk is positively associated with WTP (Adigwe, 2021; Han et al., 2021; Zhang et al., 2021). Across the study period, the number of domestic cases were below 100, while sporadic outbreaks were confined to one or two cities (Organization, 2021), therefore, the urgency and value of vaccination was not sensed by individuals living in such environment. Our findings anticipate difficulties in changing from the currently free vaccine to a required co-payment or even a complete out-of-pocket payment if most people have been vaccinated. Targeted measures to improve public awareness of COVID resurgence and the importance of vaccine effect were suggested.

The HBM theory, which was specifically developed to study preventive interventions (Orji et al., 2012; Wong et al., 2020), has illustrated that personal belief is powerful in the vaccination decision-making process (Han et al., 2021). According to the multivariate analyses, the perceived benefit of vaccination and environmental peer pressure would enhance WTP. Those who strongly agreed with the benefit of vaccination are more than two times as likely to pay for high-priced vaccines than those who strongly disagreed. Those who would accept vaccination if others took it were twice as likely to pay for high-priced vaccines relative to those immune to peers’ behavior. Perceived benefit and susceptibility were known predictors for WTP in various populations (Han et al., 2021; Harapan et al., 2020). Additionally, interventions targeting these HBM constructs have improved the effectiveness of vaccination (Jones et al., 2012; Myers, 2017). Based on our findings, it makes sense to strengthen beliefs surrounding vaccination benefits and to leverage environmental pressure.

Regarding the relation between socioeconomic factors and WTP, our study found that people with lower AHI tend to pay less for the COVID-19 vaccine. The multivariate analyses confirmed the trend, and specifically, AHI < 40k was shown to be significantly associated with the lower WTP. Urban residents were 1.57 times as likely to pay a high vaccine price. These findings were consistent with local and international studies that economically disadvantaged people were unwilling to pay irrespective of other factors (Wang et al., 2021). This highlights the need to consider the affordability of the COVID-19 vaccine, especially in low-middle-income countries. If the COVID-19 vaccine was priced at CNY 149 (the grand mean WTP in our study), over 70% of the sample and families with AHI < 210K were unwilling to pay (Figure 2; Table 2). This can be extrapolated to one billion people considering the size of Chinese population (EBoNBoSo, 2022). Domestically made vaccines have been priced at CNY 200 or 234 per dose to local governments and individual customers (Times, 2020; Author Anonymous, 2020). It seems that the COVID-19 vaccines have been over-priced and exceeded common affordability.

WTP is useful, informative evidence for a government to utilize in the provision of public goods and decision-making surrounding issues such as financing, pricing, and subsidization. In the scenario that public goods transit to private goods, WTP can inform the affordability of the public, especially in low- and middle-income countries (LMICs) where public financing is difficult. In Nigeria, only a quarter of respondents were willing to pay for vaccination, and half of the respondents were not willing to pay more than USD1.20 (Adigwe, 2021). Studies from other LMICs have reported mean WTPs of USD 30.66, 57.20, and 85.92 respectively in Malaysia, Indonesia, and Vietnam (Harapan et al., 2020; Wong et al., 2020; Vo et al., 2021), as well as WTP ranges of USD 6.81-13.62 for India and USD 49.81-68.25 for Kenya (Carpio et al., 2021; Goruntla et al., 2021). Our estimate of CNY 149, which is equivalent to USD 23.09 according to the exchange rate in 2021, has seemingly confirmed that China still belongs to the class of LMIC, and that the affordability of the population needs to be considered. On the other hand, high-income countries reported much higher mean WTPs of USD 232 and USD 318.76 for the COVID-19 vaccine in Chile and the USA respectively (Catma and Varol, 2021; Cerda and Garcia, 2021). The WTP varies greatly across the countries indicating the uneven affordability of different populations. This heterogeneity may form a barrier in the global war on the COVID-19 epidemic (Acharya et al., 2021).

Some advantages of our study were of note. The biggest advantage distinguishing this study from others could be that the validity of WTP is high. We used randomization and averaging to minimize bias. Respondents were first randomly allocated to PS or IBG method, and then further randomized to one of three IBG biding algorithms with different starting-price. The WTPs by two CVM methods were averaged and analyzed. It turned out that the IBG derived significantly higher WTPs than the PS, though methodologically expected (Frew et al., 2004). Moreover, the WTPs by two CVM methods from the same person had a poor agreement, even in extremely opposite directions observed in 10 cases. Within IBG itself, the mean WTPs were CNY 435, 329, and 309 for IBG800, IBG170, and IBG20 respectively, reflecting the “anchoring effect” inherent in this method. All these facts mean that random allocation and data averaging are necessary and have reduced the methodology-induced bias in estimating the true WTP. Another advantage is that WTP and WTV for the booster dose at the time of adequate epidemic control were rarely reported. Our study filled the gap and allowed policy-makers to keep track of WTP and WTV trajectories.

This study has some limitations. The sample size of 543 was considered small to represent the national population. In this regard, a study using Monte Carlo simulation showed that a sample size of 400 is sufficient to produce a valid WTP with little impact from the number of bidding prices (Judez et al., 2000). The representativeness may also be undermined by our sampling methods and the online-survey format. Respondents with high internet utilization may be systematically different from the general population. Snowballing sampling is limited to reaching a wide population base. Considering that two rounds of vaccination programs have been completed, and the effectiveness and safety of the vaccine are generally known to everyone, the IBG scenario did not provide data or facts about the vaccine. The social benefit of vaccination and the negative effect of non-vaccination were delineated instead. This needs to be considered when our results were compared with the early studies which were conducted before the vaccine was available. Finally, a cross-sectional study was unable to substantiate a causal relationship.

## 5 Conclusion

The willingness to vaccinate with the COVID-19 vaccine booster dose was generally high in China, especially in the younger populations. The wiliness to pay was influenced by the place of residence, vaccination status, household income, perceived benefit of vaccination, and environmental peer pressure. Study findings can inform policymakers to better design future vaccination programs and financial schemes involving out-of-pocket payments. Financial support is necessary for disadvantaged populations in view of their affordability problems.

## Data Availability

The raw data supporting the conclusion of this article will be made available by the authors, without undue reservation.
